# A Case-Control Study of Risk Factors Associated with Scrub Typhus Infection in Beijing, China

**DOI:** 10.1371/journal.pone.0063668

**Published:** 2013-05-14

**Authors:** Yanning Lyu, Lili Tian, Liqin Zhang, Xiangfeng Dou, Xiaomei Wang, Weihong Li, Xiuchun Zhang, Yulan Sun, Zengzhi Guan, Xinyu Li, Quanyi Wang

**Affiliations:** 1 Institute for Infectious Disease and Endemic Disease Prevention and Control, Beijing Center for Disease Prevention and Control, Beijing, China; 2 Department of Infectious Disease and Endemic Disease Prevention and Control, Center for Disease Prevention and Control of Pinggu District, Beijing, China; University of Malaya, Malaysia

## Abstract

To investigate the risk factors of scrub typhus infection in Beijing, China, a case-control study was carried out. Cases (n = 56) were defined as persons who were diagnosed by PCR and serological method within three years. Three neighborhood control subjects were selected by matching for age and occupation. Living at the edge of the village, living in the houses near grassland, vegetable field or ditch, house yard without cement floor, piling weeds in the house or yard, all of these were risk factors for scrub typhus infection. Working in vegetable fields and hilly areas, and harvesting in autumn posed the highest risks, with odds ratios (ORs) and 95% confidence intervals (CIs) of 3.7 (1.1–11.9), 8.2 (1.4–49.5), and 17.2 (5.1–57.9), respectively. These results would be useful for the establishment of a detail control strategy for scrub typhus infection in Beijing, China.

## Introduction

Scrub typhus, also known as tsutsugamushi disease, is an acute, febrile infectious illness. It is caused by Orientia tsutsugamushi (formerly named as Rickettsia tsutsugamushi), a gram-negative obligate intracellular bacterium. Scrub typhus can be transmitted to human and other vertebrates by the bite of an infected larval trombiculid mite (chigger), which belongs to the family Trombiculidae and primarily consisting of the genus Leptortrombidium [1]. This disease is widely endemic in a geographically confined area of Asia-Pacific region, the so-called tsutsugamushi triangle from northern Japan and far-eastern Russia in the north to northern Australia in the south and to Pakistan and Afghanistan in the west, as well as in the islands of the western Pacific and Indian Oceans. More than half (55%) of the world’s population live in the areas where scrub typhus is endemic. An estimated one million cases occur annually [2]. Scrub typhus accounts for 20% of all febrile episodes in the endemic areas [3] and is a sporadic problem during training of military personnel [4], [5]. Countries not endemic for this disease have also reported cases involving visitors to the disease-endemic-areas [6]–[8]. Scrub typhus has been known in southern China for thousands of years [9]. However, the disease has emerged in northern China only in the last three decades [10], such as Hebei Province, Tianjin City, Shandong Province, Jilin Province and so on. There was an outbreak of scrub typhus in the fall of 2008 in the northeast area of Beijing [11], [12], where the disease had not been known to occur previously.

Most cases in disease-endemic areas occur through agricultural exposure such as working in rice fields in South Korea, Thailand, and Japan, and in oil palm and rubber plantations in Malaysia [13]. The most typical complaints of infected persons are fever, headache, rash, eschar, and lymphadenopathy [14]. In some cases, central nervous system symptoms or circulatory collapse caused by disseminated intravascular coagulation can occur, especially in patients with delayed diagnosis [15]. The clinical course of the disease and the prognosis vary depending on the character of the endemic strain [16], [17]. Patients with scrub typhus have high fatality rates of up to 50–60% and clinical courses ranged from 10 to 28 days before antibiotics were introduced [13], [18]. Scrub typhus is usually successfully treated with doxycycline, tetracycline, or chloramphenicol, while repellents and chemoprophylaxis have limited efficacy.

Factors that may contribute to the increase of scrub typhus include: increase outdoor activities performed by urban inhabitants, rapid urbanization of societies, aging populations, changes in ecological environment, development of ecotourism, significant evolution of diagnostic techniques and public surveillance systems [19]–[21]. Scrub typhus is a public health issue in Asia, where one million new cases are identified annually and one billion people may be at risk for the disease [22]. In China, scrub typhus was mainly endemic in the southern region of Yangtze River before 1985. Since the outbreak in Shandong province in 1986, the epidemic focus of scrub typhus had moved quickly to the north China. Ever since, increasing number of cases were reported in north China, such as Hebei, Shandong, Henan, Tianjin and Beijing etc. With the emergence of antibiotic resistant strains and prevalence of eco-tourism, scrub typhus prevention and control has become an important public health concern [23].

Although some studies of scrub typhus have been performed in disease endemic areas, little attention has been given to the living environment, living habits and behavioral risk factors of the disease. Scrub typhus is not transmitted directly from person to person; it is only transmitted by the bites of vectors. Therefore, the human living habits and health behaviors during outdoor activities are meaningful factors for strategies to control scrub typhus. To identify the kinds of living and behavioral risk factors associated with the incidence of scrub typhus, we undertook a case-control study in Beijing.

## Materials and Methods

### Ethics Statement

The Institutional Review Board (IRB) of Beijing Center for Diseases Prevention and Control reviewed and approved all protocols to conduct the Scrub typhus study entitled “A Case-control Study of Risk Factors Associated with Scrub Typhus Infection in Beijing, China”. Written and informed consent was obtained from all patients involved in this study.

### Study Design and Participants

The study area consists of 15 villages and towns that are located in the northeast of Beijing, situated at the junction of Beijing, Tianjin and Hebei province, with 1075 km^2^ area and a population of 500,000. This district has shown a high incidence of scrub typhus in recent years. The prevalence rate of scrub typhus in this area was 0.029‰ in 2009 and 0.109‰ in 2010 according to the report of China Information System for Diseases Control and Prevention.

The case inclusion criteria were scrub typhus cases reported in the China Information System for Diseases Control and Prevention within the past three years. The case exclusion criteria were the reported cases that lacked laboratory confirmation (a four-fold increase in the IgG antibody titer in follow-up serum, positive IgM antibody detection or genetic material detected using polymerase chain reaction [11]). Eligible controls were defined as inhabitants living (more than 6 months) in the same villages or towns as the case subjects, who were matched for age (within 5 years) and occupation, and lacked a history of scrub typhus within three years according to the China Information System for Diseases Control and Prevention. The scrub typhus IgG test of these controls was negative. A total of 56 cases were recruited from the 15 study areas during October and December 2011. The distribution of 56 cases in these 15 villages and towns were uneven. One town has 14 cases, one town has 10 cases. Two towns have 5 cases each, two towns have 4 cases each, two towns have 3 cases each, one town has 2 cases, and six towns have one case each. All these 56 cases were diagnosed positive by serological method, and 47 cases of them were diagnosed positive by PCR method. And 168 identified matched controls (1∶3 pair matching) from their nearest neighbors were recruited at the same time.

### Data Collection

56 cases of scrub typhus from 2008 to 2010 were selected. Trained interviewers visited the cases and controls. We were particularly interested in the residential environment, living habits and modifiable behaviors during outdoor activities, such as the type of agricultural work, as well as feeding animal activities. The potential confounders of age and occupation (farming and non-farming) were controlled by matching. A standardized questionnaire was used by trained interviewers to collect detailed information of cases and controls. Three main categories of variables were considered in the questionnaire: socioeconomic status (age, education level, and residential environment), living habits and outdoor activities (work places, type of work engaged in within the previous one month in consideration of the incubation period and delay-time from disease onset).

We obtained verbal informed consent from all study subjects. This study was reviewed and approved by the Beijing Centers of Disease Control and Prevention (BJCDC), the governmental agency in charge of communicable disease control in Beijing.

### Statistical Analysis

EpiData software was used to develop the data set. Statistical analyses were performed with SPSS 13.0 software (SPSS Inc., Chicago, IL, USA). The multivariate logistic regression model was used to investigate the potential correlative factors that were associated with scrub typhus infection. The significance level for entering the multivariate logistic regression model was set as 0.10, and the level for staying in the model was 0.05. A *P* value of <0.05 was considered statistically significant.

## Results

### General Information of the Study Subjects

We recruited 56 scrub typhus cases and 168 matched controls. For cases and controls, respectively, 48.2% and 66.1% were female, 67.9% and 78.6% were farmers. The greatest age class in the age distribution was the 40∼ years group, and this was similar between the two groups. There were no significant differences in some demographic characteristics, such as occupation, age and education years between case group and control group (Table 1). When matched with the same age and occupation, it is very difficult to find the controls of the same gender who living in the neighborhood in this area. And according to the report before, there is no significant difference in the prevalence of scrub typhus in this area between male and female [12], [24]. Therefore, in this instance, gender was not chosen as a match factor.

**Table 1:** General information of the scrub typhus cases and controls enrolled in the study.

Characteristic	Case (n = 56)		Control (n = 168)		*P value*
	No.	%	No.	%	
Age group, years					0.620
20∼	6	10.7	24	14.3	
40∼	35	62.5	93	55.4	
60∼	15	26.8	51	29.8	
Gender					0.017
Male	29	51.8	57	33.9	
Female	27	48.2	111	66.1	
Occupation					0.128
Farmer	38	67.9	132	78.6	
Non-farmer	18	32.1	36	21.4	
Education, years					0.533
≤6	26	46.4	70	41.7	
> 6	30	53.6	98	58.3	

### Living Environment

For cases and controls, respectively, 98.2% and 92.9% lived in houses, others lived in apartments, 69.6% and 82.1% lived in houses with yard of cement floor, 51.8% and 17.3% live in houses near grassland, vegetable field or ditch, 14.3% and 1.8% piled weeds in the room, and 60.7% and 9.6% piled weeds in the yard. There were significant differences in most factors of living environment, except housing type between cases and controls (Table 2).

**Table 2:** Living environment of the scrub typhus cases and controls enrolled in the study.

Living environment	Case (n = 56)		Control (n = 168)		*P value*
	No.	%	No.	%	
Residential location					<0.001
Edge of village	32	57.1	32	19.0	
Center of village and town	24	42.9	136	81.0	
Housing type					0.138
House	55	98.2	156	92.9	
Apartment/others	1	1.8	12	7.1	
House yard with cement floor					0.047
Yes	39	69.6	138	82.1	
No	17	30.4	30	17.9	
Houses near grassland, vegetable field or ditch					<0.001
Yes	29	51.8	27	17.3	
No	27	48.2	141	82.7	
Piling weeds in the house					0.001
Yes	8	14.3	3	1.8	
No	48	85.7	165	98.2	
Piling weeds in the yard					<0.001
Yes	34	60.7	16	9.6	
No	24	39.3	152	90.4	

### Places of Work and Type of Outdoor Activities

Table 3 shows the ORs for the work places and type of outdoor activities within one month from the onset using single factor logistic regression analysis. Persons who had worked in farmlands, vegetable fields, and vinyl house within one month had significantly higher risk than other persons. The ORs (95% CI) for farmlands, vegetable fields and vinyl house were 3.8 (2.0–7.2), 2.0 (1.0–3.0), and 6.4 (1.1–35.9) respectively. Persons who had engaged in harvesting in autumn and other outdoor activities, such as picking wild fruit, collecting firewood had a significantly higher risk of scrub typhus infection with ORs (95% CI) of 4.6 (2.4–8.8) and 2.0 (1.1–3.7). Leisure activities such as camping, walking and resting on grassland were positively associated with infection risk and had an OR (95% CI) of 2.1 (1.0–4.2).

**Table 3:** Associations between risk factors in outdoor activities and scrub typhus, Beijing.

Exposure within one month	Case (n = 56) No. (%)	Control (n = 168)† No. (%)	OR (95% CI)*
Places of work			
Farmland	38 (67.9)	60 (35.7)	3.8 (2.0∼7.2)
Vegetable fields	19 (33.9)	34 (20.2)	2.0 (1.0∼3.0)
Vinyl house	4 (7.1)	2 (1.2)	6.4 (1.1∼35.9)
Woodland and hilly area	22 (39.3)	45 (26.8)	1.8 (0.9∼3.3)
Raising animals‡	36 (64.3)	73 (43.5)	2.3 (1.3∼4.4)
Working in the field	89 (53.0)	44 (78.6)	3.3 (1.6∼6.6)
Harvesting in autumn	38 (67.9)	53 (31.5)	4.6 (2.4∼8.8)
Sowing	22 (39.3)	45 (26.8)	1.8 (0.9∼3.3)
Other outdoor activities (Picking wild fruit, collecting firewood)	25 (44.6)	49 (29.2)	2.0 (1.1∼3.7)
Leisure activities§	17 (30.4)	29 (17.3)	2.1 (1.0∼4.2)

* OR = odds ratio; CI = confidence interval.

† Matched by age and occupation.

‡ Raising animals involved rabbit, cat, dog, pig, cattle, sheep, goat and poultry.

§ Leisure activities involved camping, walking and resting on a grass land.

### Multivariate Logistic Regression Analysis of Risk Factors

Backward selection logistic regression analysis showed that when using gender, residential location, housing type, working places, activity type and living habits etc. as independent variables, and scrub typhus infection as a dependent variable, the model had significance, p<0.001, R^2^ = 0.636. The results showed that gender, education≤ 6 years, working in vegetable fields, hilly area, harvesting in autumn, living in a house without cement floor, piling weeds in the house and yard, living in houses near grassland, vegetable field or ditch were positively associated with scrub typhus infection. The details are showed in Table 4.

**Table 4:** Multivariate logistic regression analysis of risk factors for scrub typhus infection, Beijing*.

Item	Partial regressioncoefficient	Partial regression coefficientstandard error	*P value*	AdjustedOR	Adjusted OR95.0% CI
Gender	0.987	0.494	0.046	2.7	1.0∼7.1
Education≤ 6 years	2.773	1.104	0.012	16.0	1.8∼139.2
Working in vegetable fields	1.302	0.601	0.030	3.7	1.1∼11.9
Working in woodland and hilly area	2.101	0.919	0.022	8.2	1.4∼49.5
Harvesting in autumn	2.842	0.621	<0.001	17.2	5.1∼57.9
Sowing	−1.414	0.611	0.021	0.2	0.1∼0.8
House yard without cement floor	1.437	0.711	0.043	4.2	1.0∼17.0
Piling weeds in the house and yard	2.669	0.566	<0.001	14.4	4.8∼43.7
Living in houses near grassland, vegetable field or ditch	1.906	0.543	<0.001	6.7	2.3∼19.5
Raising animals	−0.938	0.550	0.088	0.4	0.1∼1.2
Constant	−45.743	2.188*10^4^	0.998	<0.0	–

* The significance level for entering the multivariate logistic regression model was set as 0.10 and for staying in it was set as 0.05.

* Negatively correlated factors: Sowing.

* Positive correlation factors: Gender, education≤ 6 years, working in vegetable fields, working in hilly area, harvesting in autumn, house yard without cement floor, piling weeds in the house and yard, living in houses near grassland, vegetable field or ditch.

## Discussion

Since the emergence of the first scrub typhus infection case in 2008 in northeast district of Beijing, an increasing number of cases were reported from 2009 to 2011 in Beijing [11], [12], [24]. Recently, Beijing has been confirmed as the natural foci of scrub typhus for the first time by Beijing Center for Disease Prevention and Control laboratory (data not shown). 56-kDa type-specific antigen gene DNA sequencing revealed that the prevalent stain of Orientia tsutsugamushi in this area was identical with a homology of 96% with Kawasaki type [11]. In order to find out the risk factors associated with this disease, a case-control study was carried out. In this study, we examined the association between scrub typhus infection and living environment, living habits, work-related behavioral factors and outdoor activities. The results would provide the theoretic basis for setting up the prevention and control measures. To our knowledge, few comparable studies were carried out in China to investigate the risk factors associated with the scrub typhus infection. Our work focused on the specific regional details, such as living environment, house typing and outdoor activities etc, which provided results that could be more valuable for establishing the effective prevention and control strategy for Beijing or even northern area of China.

The results showed that comparing with living in town or in the center of the village, living at the edge of the village, living in the houses near grassland, vegetable field or ditch, were positively associated with scrub typhus infection. The house at the edge of the village is more likely near the farmland or grassland, where the chigger mite grows, and this makes resident contact with transmission vectors of scrub typhus easily. Our results also showed that living in a house that has a yard without cement floor, or piling weeds in the house or yard was a high risk factor of scrub typhus infection. The lack of the cement floor in the house yard allows grass or weeds to grow easily, and these weeds in the house and yard area can increase the risk for people to come into contact with the transmission vector. All these factors increase the exposure and resident contact with transmission vectors of scrub typhus.

Although farmers are considered to be a high-risk group for scrub typhus [13], [18], the association has not been clarified in detail. Our study showed that scrub typhus infection was strongly and positively associated with working in fields and harvesting in autumn, but sowing is negatively associated with it. Leptotrombidium scutellare, the predominant transmission vector in north China [25], 26, begins to appear in September and has a peak population in October and November. In Beijing, most of the sowing activities are carried out in spring, which could explain why sowing is not a risk factor for scrub typhus infection in Beijing.

Farm and forest workers are well known to be high-risk groups for scrub typhus. We found strong positive associations with working in the vegetable field, woodland and hilly area; persons who had harvested crops or had gathered wood or weeds in the field within the previous month also showed a high risk for scrub typhus infection.

Our results suggest that people who tend to expose their skin during outdoor activities are vulnerable to scrub typhus infection. As the proportion of people raising animals was low, the possession of pets or domestic animals was not associated with the disease. In our study, we found that only 2.3% and 3.3% of the participants (cases and control respectively) had received health education for scrub typhus during their lifetimes. Therefore, the efficacy of these measures for preventing scrub typhus was not clearly evaluated. Beijing Center for Disease Prevention and Control should begin to perform an intensive health education program in disease-endemic areas. And a prospective study is required in these regions to investigate the efficacy of the prevention measures.

According to the report of the of China Information System for Diseases Control and Prevention, the difference in ratio of male and female scrub typhus patients is not significant, but the multivariate logistic regression analysis showed that male has a higher risk of catching the disease than female. Typically, men are more involved in the farm work and field work than women in north China, and this offers more chance for male to get the chigger bite than female, and thus a higher risk to catch the scrub typhus.

The multivariate logistic regression analysis also showed that lower education level (education ≤ 6 years) is a risk factor for scrub typhus infection in this area. Higher education provided individuals with more knowledge about this disease and allowed easier access to get health education about this disease through various channels. Individuals with higher education may also possess better personal hygienic habits, such as changing clothes or taking bath after field work. All these behaviors can help to protect a man from the chigger bite. Since these contents were not included in the questionnaire, we need to carry out further study to confirm the hypothesis in the future.

Our study has specific strengths. First, the study was representative of the Beijing population and specifically designed according to the rural conditions of Beijing area. Second, the results have definite implications for public health because the study targeted modifiable behaviors.

There were also limitations to our study. First, the sample size was relatively small considering the heterogeneity of the study participants. Second, there were some potential for recall bias because of using cases that had the infection in 2008 and 2009, which involved a longer time from the onset to be interviewed.

Despite these limitations, our study presents worthy results that can contribute to the establishment of an evidence-based intervention strategy to reduce the incidence of scrub typhus. Outdoor activities and living habits can make a person vulnerable to scrub typhus infection. Conducting health education constantly and increasing public awareness, such as changing living habits of not piling weeds in the house and yard, improving living conditions to the best of one’s ability, cementing the ground of the house and yard to reduce the weed growth, taking preventive behaviors that reduce exposure to infected mites, e.g., wearing a long-sleeve shirt, not resting on the bare field or grass may have significant effects in preventing scrub typhus infection in Beijing.

In conclusion, our findings will be helpful in the establishment of a detailed intervention program to control scrub typhus in Beijing area or other similar disease-endemic area in China.

## References

[1] Phongmany S, Rolain JM, Phetsouvanh R, Blacksell SD, Soukkhaseum V, et al (2006) Rickettsial infections and fever, Vientiane, Laos. Emerg Infect Dis 12: 256–262. 16494751 10.3201/eid1202.050900 PMC3373100

[2] Rosenberg R (1997) Drug-resistant scrub typhus: Paradigm and paradox. Parasitol Today 13: 131–132. 15275098 10.1016/s0169-4758(97)01020-x

[3] Brown GW, Robinson DM, Huxsoll DL, Ng TS, Lim KJ (1976) Scrub typhus: a common cause of illness in indigenous populations. Trans R Soc Trop Med Hyg 70: 444–448. 402722 10.1016/0035-9203(76)90127-9

[4] Bavaro MF, Kelly DJ, Dasch GA, Hale BR, Olson P (2005) History of U.S. military contributions to the study of rickettsial diseases. Mil Med 170: 49–60. 15916283 10.7205/milmed.170.4s.49

[5] Cao M, Guo H, Tang T, Wang C, Li X, et al (2006) Spring scrub typhus, People's Rrepublic of China. Emerg Infect Dis 12: 1463–1465. 17073108 10.3201/eid1209.060257 PMC3294749

[6] Watt G, Strickman D (1994) Life-threatening scrub typhus in a traveler returning from Thailand. Clin Infect Dis 18: 624–626. 8038320 10.1093/clinids/18.4.624

[7] Jensenius M, Montelius R, Berild D, Vene S (2006) Scrub typhus imported to Scandinavia. Scand J Infect Dis 38: 200–202. 16500780 10.1080/00365540500277342

[8] Jensenius M, Davis X, von Sonnenburg F, Schwartz E, Keystone JS, et al (2009) Multicenter GeoSentinel analysis of rickettsial diseases in international travelers, 1996–2008. Emerg Infect Dis 15: 1791–1798. 19891867 10.3201/eid1511.090677 PMC2857242

[9] Fan MY, Walker DH, Yu SR, Liu QH (1987) Epidemiology and ecology of rickettsial diseases in the People's Republic of China. Rev Infect Dis 9: 823–840. 3326129

[10] Yu C (1992) Report on first finding an epidemic of Scrub typhus in north rural areas. Zhonghua Liu Xing Bing Xue Za Zhi 13: 212–215. 1301265

[11] Fu XP, Liu YY, Zhang BH, Wang JQ, Zhang JS, et al (2011) Laboratory confirmation of scrub typhus outbreak in Pinggu district, Beijing. Chin J Vector Biol & Control 22: 137–140.

[12] Zhao YH, Xue H (2010) Epidemiology Characteristic of 15 Cases of Scrub Typhus in Pinggu District of Beijing. Occup and Health 26: 2991–2992.

[13] Silpapojakul K (1997) Scrub typhus in the Western Pacific region. Ann Acad Med Singapore 26: 794–800. 9522982

[14] Lewis MD, Yousuf AA, Lerdthusnee K, Razee A, Chandranoi K, et al (2003) Scrub typhus reemergence in the Maldives. Emerg Infect Dis 9: 1638–1641. 14720413 10.3201/eid0912.030212 PMC3034347

[15] Yi KS, Chong Y, Covington SC, Donahue BJ, Rothen RL, et al (1993) Scrub typhus in Korea: importance of early clinical diagnosis in this newly recognized endemic area. Mil Med 158: 269–273. 8479637

[16] Ebisawa II (1995) Current Epidemiology and Treatment of Tsutsugamushi Disease in Japan. J Travel Med 2: 218–220. 9815394 10.1111/j.1708-8305.1995.tb00662.x

[17] Kim DM, Yun NR, Neupane GP, Shin SH, Ryu SY, et al (2011) Differences in clinical features according to Boryoung and Karp genotypes of Orientia tsutsgamushi. PloS One 6: e22731. 21857951 10.1371/journal.pone.0022731 PMC3156117

[18] Kelly DJ, Richards AL, Temenak J, Strickman D, Dasch GA (2002) The past and present threat of rickettsial diseases to military medicine and international public health. Clin Infect Dis 34: S145–S169. 12016590 10.1086/339908

[19] Strickman D, Tanskul P, Eamsila C, Kelly DJ (1994) Prevalence of antibodies to rickettsiae in the human population of suburban Bangkok. Am J Trop Med Hyg 51: 149–153. 8074248 10.4269/ajtmh.1994.51.149

[20] Matsui T, Kramer MH, Mendlein JM, Osaka K, Ohyama T, et al (2002) Evaluation of National Tsutsugamushi Disease Surveillance–Japan, 2000. Jpn J Infect Dis 55: 197–203. 12606829

[21] Lee YS, Wang PH, Tseng SJ, Ko CF, Teng HJ (2006) Epidemiology of scrub typhus in eastern Taiwan, 2000–2004. Jpn J Infect Dis 59: 235–238. 16936341

[22] Watt G, Parola P (2003) Scrub typhus and tropical rickettsioses. Curr Opin Infect Dis 16: 429–436. 14501995 10.1097/00001432-200310000-00009

[23] Watt G, Chouriyagune C, Ruangweerayud R, Watcharapichat P, Phulsuksombati D, et al (1996) Scrub typhus infections poorly responsive to antibiotics in northern Thailand. Lancet 348: 86–89. 8676722 10.1016/s0140-6736(96)02501-9

[24] Tian LL, Zhang XC, Guan ZZ, Li WH, Dou XF, et al (2011) Epidemiology of 67 scrub typhus cases in Beijing. Disease Surveillance 26: 211–213.

[25] Wu GH (2005) The Study on the vector of Tsutsugamushi Disease - Chigger Mite in China. Chin J Vector Bio & Control 16: 485–487.

[26] Su JJ, Wang Y, Zhou J, Yu B, Yang ZQ (2012) Advances in research of tsutsugamushi disease epidemiology in China in recent years. Chin J Hyg Insect & Equip 18: 160–163.

